# Mésusage des dermocorticoïdes: une image vaut mille mots

**DOI:** 10.11604/pamj.2019.33.24.18273

**Published:** 2019-05-14

**Authors:** Asmaa Sqalli Houssaini, Badreddine Hassam

**Affiliations:** 1 Service de Dermatologie et Vénérologie, Centre Hospitalier Universitaire Ibn Sina, Faculté de Médecine et de Pharmacie, Université Mohammed V, Rabat, Maroc

**Keywords:** Dermocorticoïdes, atrophie cutanée, auto-médication, Dermocorticoids, skin atrophy, self-medication

## Abstract

Dermocorticoids (DC) are the first line treatment for mild to moderate psoriasis. Despite their relative safety, long-term use and most of all self-medication practice could expose them to a risk of skin atrophy. We report the case of a 50-year old female patient, treated for psoriasis with chronic application of topical dermocorticoids over several years. Her skin appeared to be very thin, displaying the underlying venous network, telangiectasias as well as stretch marks which are manifestations of dermo-epidermal atrophy. Skin atrophy is the most common adverse effect of dermocorticoids and it is due to their strong antiproliferative effect on keratinocytes, melanocytes and fibroblasts which synthesize the collagen. It may be associated with delayed wound healing and various infectious complications; hence the importance of providing proper patient education.

Les dermocorticoïdes (DC) constituent le traitement de première intention du psoriasis léger à modéré. Malgré leur relative innocuité, l'utilisation à long terme et surtout par auto-médication pourrait exposer à un risque d'atrophie cutanée. Nous rapportons le cas d'une patiente de 50 ans, suivie pour psoriasis depuis plusieurs années avec application chronique de DC, qui présentait une peau très fine laissant apparaitre le réseau veineux sous-jacent, des télangiectasies, ainsi que des vergetures témoignant de l'atrophie dermo-épidermique. L'atrophie cutanée est l'effet indésirable des DC le plus fréquent; elle s'explique par leur effet antiprolifératif sur les kératinocytes, les mélanocytes et les fibroblastes qui synthétisent le collagène. Elle s'accompagne de retard de la cicatrisation et de diverses complications infectieuses d'où l'importance de l'éducation thérapeutique.

**Figure 1:**
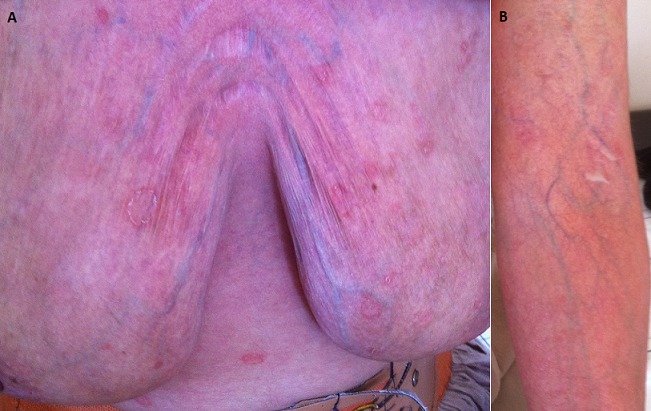
A) peau atrophiée, telengiectasies, vergetures au niveau du thorax; B) peau atrophiée, telengiectasies, vergetures au niveau du bras

